# An Experimentally Aided Operational Virtual Prototyping to Obtain the Best Spindle Speed during Face Milling of Large-Size Structures

**DOI:** 10.3390/ma14216562

**Published:** 2021-11-01

**Authors:** Krzysztof J. Kaliński, Marek A. Galewski, Michał R. Mazur, Natalia Stawicka-Morawska

**Affiliations:** Faculty of Mechanical Engineering and Ship Technology, Gdansk University of Technology, 80-233 Gdansk, Poland; krzysztof.kalinski@pg.edu.pl (K.J.K.); michal.mazur@pg.edu.pl (M.R.M.); natalia.morawska@pg.edu.pl (N.S.-M.)

**Keywords:** face milling, cutting process dynamics, vibration suppression, hybrid modeling, modal analysis, virtual prototyping

## Abstract

The paper presents an original method concerning the problem of vibration reduction in the general case while milling large-size and geometrically complex details with the use of an innovative approach to the selection of spindle speed. A computational model is obtained by applying the so-called operational approach to identify the parameters of the workpiece modal model. Thanks to the experimental modal analysis results, modal subsystem identification was performed and reliable process data for simulation studies were obtained. Next, simulations of the milling process, for successive values of the spindle speed, are repeated until the best vibration state of the workpiece is obtained. For this purpose, the root mean square values of the time plots of vibration displacements are examined. The effectiveness of the approach proposed for reducing vibrations in the process of face milling is verified on the basis of the results of appropriate experimental investigations. The economic profitability of the implementation of the operational technique in the production practice of enterprises dealing with mechanical processing is demonstrated as well.

## 1. Introduction

According to Fei et al. [1], dynamic phenomena during milling operations of large-size structures are manifested primarily in the relative vibrations of the tool and the workpiece. Hao et al. noted [2] that they impose significant restrictions on the further increase in productivity and product quality. In order to increase machining efficiency, Płodzień et al. proposed a new method called high feed milling [3]. However, its use results in a significant increase in the cutting force components and, as a consequence, the vibration level and deterioration of the surface roughness. Under certain circumstances, the growing vibrations can lead to instability, and then to self-excited vibrations referred as *chatter*, which was emphasized, for example, by Quintana and Ciurana [4], Mane et al. [5], and López de Lacalle et al. [6]. *Chatter* greatly reduces processing efficiency, worsens the machined surface (Urbikain et al. [7]), and may even lead to the destruction of tools and workpieces (Nouari et al. [8]). Although the appearance of *chatter* vibrations worsens surface finish and tool life rapidly decreases or may result in its destruction, few references in the literature seek the relationship between the level of controlled vibrations and machining accuracy as well as the durability of the cutting edges, e.g., by Li et al. [9].

The problem of *chatter* vibration prevention has been addressed in various ways. For example, Quintana and Ciurana [4] presented the state of research of *chatter* in the machining process and classified the existing methods that ensure stable cutting conditions. Similarly, Munoa et al. [10] critically reviewed the evolution of the different *chatter* suppression techniques. Yue et al. [11] presented the survey of methods and Zhu et al. [12] illustrated the current state-of-the-art regarding the problems of *chatter* prediction and detection in milling processes. The study of *chatter* stability of special milling tools such as variable pitch and crest-cut cutters was presented by Tehranizadeh et al. [13]. Liu and Zhou [14] described the research overview of *chatter* stability during the milling of thin-walled parts. In particular, it applied to the milling of thin floors considered by Campa et al. [15] and Dang et al. [16]. Yang et al. [17] focused on the efficient decomposition-condensation method to predict *chatter* in the in-process workpiece dynamics when applied to thin-walled parts. Liu et al. [18] presented a position-oriented process monitoring model based on correlation of the cutting position with cutting force, acceleration, and spindle power to improve the machining quality and efficiency. In [19], Ren et al. obtained *chatter* stability by application of the semi discretization method based on the integrated vibration model and the cutting dynamics. In [20], Artetxe et al. presented a cutting force prediction model that included tool runout and workpiece flexibility. However, the effective use of the above proposed developments is facing serious obstacles because of the interference with the structure of the machine. The latter especially concerns a wide group of active damping methods, such as application of the optimal control for *chatter* mitigation in milling (Monnin et al. [21]), and the use of the own drives of a large milling machine to suppress *chatter,* together with an external accelerometer located close to the center point of the tool (Munoa et al. [22]). Feng et al. [23] considered the varying stiffness of the milling system. Wan et al. [24] analyzed damping of milling *chatter* vibration by means of a contactless electromagnetic actuator with two degrees of freedom integrated in the spindle system, while Moradi et al. [25] displayed suppression of regenerative *chatter* in nonlinear milling processes. The other group obeys semi-active methods, e.g., sliding mode control and an electromagnetic actuator for which the velocity state in a closed-loop control with feedback is not needed (Wan et al. [26]), usage of an electromagnetic spring with adjustable negative stiffness in semi-active control (Pu et al. [27]), or replacing the mass element with an inerter in a classic passive damper (Wang et al. [28]). Díaz-Tena et al. [29] applied magnetorheological fluids to reduce vibrations when milling thin floor elements in high-speed machining under conditions where there is instability at theoretically optimal cutting parameters. Muhammad et al. [30] reviewed the state-of-the-art on the control of machining *chatter* vibrations, including damping methods related to boring, turning, and milling processes. Besides, we observe usage of a magnetorheological damper device to modify the stability boundaries with a significant increase in the productivity factor of thin-floor components (Puma-Araujo et al. [31]), as well as vibration-assisted machining (Chen et al. [32]). In real applications, much more sophisticated techniques and algorithms for *chatter* detection can be used. Thus, Albertelli et al. [33] developed an algorithm to be implemented as real-time *chatter* detection in industrial conditions or even as a module of a vibration control system. Yao et al. [34] dealt with real-time *chatter* detection and automatic suppression as well, but concerning intelligent spindle systems. Yang et al. [35] disclosed that the approximate entropy and the sample entropy were applicable to continuous and intermittent *chatter* detection in milling. Caliskan et al. [36] presented an online *chatter* detection method by monitoring the increasing vibration energy of non-periodic components. Aside from being technologically advanced, they are often costly and thus have limited use in industrial conditions.

The methodology of vibration suppression through spindle speed variation in high-speed milling by slender tools was successfully applied by Song et al. [37] and Urbikain et al. [38]. The efficiency of the modified method of vibration suppression in the potentially unstable regions of spindle speeds, resulting from the position of stability lobes, was confirmed by Kaliński and Galewski [39]. Vibration suppression using the method of spindle speed variation, however, is less effective in the case of milling flexible structures. Thus, the further developments led to the minimization of the cutting forces’ work along the direction of the corresponding cutting layer thickness, as illustrated by Kaliński and Galewski [40], as well as by Kaliński [41]. Subsequently it should also be noted (Kaliński et al. [42]) that this method may not offer the possibility of operating within the full range of spindle speeds of the machine tool. Moreover, methods focusing solely on the reduction of *chatter* overlook other dynamic phenomena of more significant importance in the machining of large-size workpieces, e.g., forced vibrations, although, according to Uriarte et al. [43], the *chatter* vibration phenomenon cannot be treated as the only challenge for the development of vibration suppression methods.

The above proves that only simulations of the computational model of the large-size machining process, taking into account the complex state of vibrations at the same time, are a reasonable means leading to an effective solution. Examples of simulation techniques used so far include the following:

- Virtual time simulation i.e., virtual prototyping (VP). During the simulation, a simplified model of the tool (all cutting teeth are exactly the same) and the workpiece is assumed, and some effects (appearing in the experiment) are ignored (for example, the effects of balancing and tool bending considered by Totis et al. [44]). In order to improve the validity of the calculation model, Kaliński et al. [45] proposed a technique of the experimentally aided virtual prototyping (EAVP);
- Hardware in the loop simulation (HiLS) used by Kaliński and Galewski [46] to emulate at least a part of a controlled mechanical structure or process, and the rest remained a material structure. The HiLS technique allows us to speed up the process of creating a control system and software;
- Hardware simulation of the dynamics of the milling process, used by Yao et al. [47], Fu et al. [48], and Kuljanic et al. [49] to develop a device that detects *chatter* vibrations. An appropriate model of the workpiece, tool, and cutting process should be derived. Then, proper programming and optimization techniques must be applied to fulfill real-time (RT) constraints. Moreover, hardware solutions, especially based on field programmable gate array (FPGA), may be required to achieve a better response time and better system reliability and repeatability (Mazur et al. [50], Rodríguez-Andina et al. [51]). In some solutions, e.g., developed by Fayose [52], mechanical systems are simulated with the use of analog electronics;
- The combination of software programming techniques with reconfigurable input/output architecture and real-time FPGA capabilities, proposed by Urbikain et al. [53]. A personal application specifically geared towards machining simulation allows you to simultaneously record the following: machining forces, accelerations, noise, and/or sound pressure. The LabVIEW FPGA module has compiled the LabVIEW VI to the FPGA hardware;
- The combination of HiLS techniques with the advantages of real-time FPGA simulation, enabling the construction of an HiLS system and taking into account very tight time constraints, as suggested by Naets et al. [54]. FPGA-based RT systems have very short response times and low *jitter* values that can be limited to a few nanoseconds.

As there is no explicit control signal in the expected milling simulation process of large-size workpieces, thanks to which there is no need to operate in the real-time domain, the technique of experimentally aided operational virtual prototyping (EAOVP) was used in the newly presented method. It was developed as a result of a creative and significant modification of the EAVP technique, previously elaborated by Kaliński et al. [45]. The main differences and advantages of the introduced modifications, compared with the existing technique, are as follows:

- Elimination of the labor-intensive and time-consuming stage of assessing the compliance of the finite element model (FEM) with the real object, because this stage greatly extends the process of creating a computational model, which is then subjected to simulation;
- Simplification of the previously adopted computational model of a rigid milling tool, in accordance with the convention of the rigid finite element method (RFEM), briefly and synthetically described by Kaliński [41], and taking into account the dynamics of the workpiece changing over time;
- Simultaneously ensuring the cost-effectiveness of the proposed approach without losing the required machining accuracy.

## 2. Simulation Model

### 2.1. Cutting Process Dynamics

The subject of the considerations is the process of face milling of a flexible workpiece with a multi-edge milling cutter. The dynamics of the machining process was analyzed with the following assumptions (Kaliński [41], Kaliński et al. [45]):

- The tool fixed in the holder, rotating with the desired spindle speed *n*, and the workpiece mounted on the table, moving with the desired feed speed *v_f_*, are the only ones taken into account. The influence of the remaining parts of the milling machine on the dynamics of the machining process can be neglected (Fei et al. [1], Uriarte et al. [43]).
- The flexibility of the workpiece, which characterizes the machining of large-size flexible elements on multi-axis machining centers, was taken into account (Fei et al. [1], Kaliński et al. [45]).
- For modeling the dynamics of the cutting process, coupling elements (CEs) were adopted, which were located at the conventional contact points of the tool edges with the workpiece (Kaliński [41]). The momentary positions of the tips of the cooperating edges of the rotating tool were assumed as these points.
- The passage of the current edge along the cutting layer causes a proportional feedback, and the passage of the previous edge additionally causes a delayed feedback. Thanks to this, it is possible to consider the effect of multiple trace regeneration in the calculation model.

**Figure 1:**
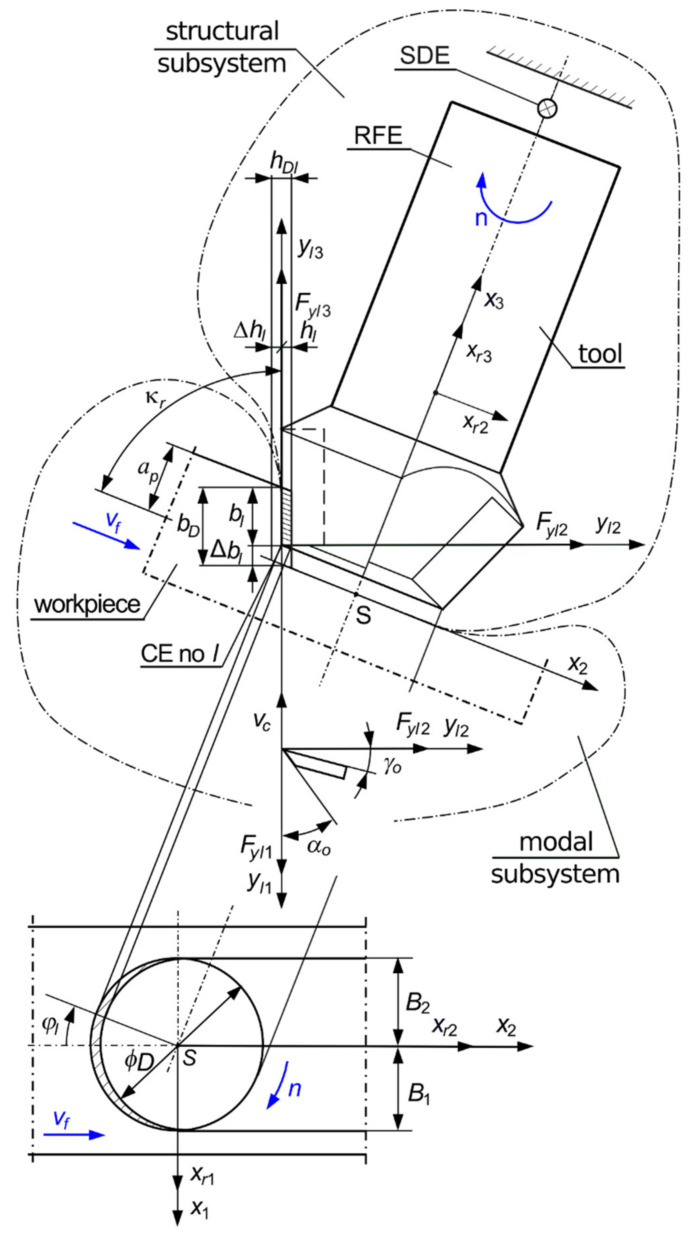
Scheme of a face milling of a flexible workpiece.

As a result of modeling the dynamics of the milling process, a system was obtained (Figure 1) consisting of the following: a rigid body called the rigid finite element (RFE), connected to a support (tool holder) by means of the spring damping element (SDE) (Kaliński [41]); a stationary model mapping the structure of a flexible workpiece; and coupling elements (CEs), the positions of which correspond to the instantaneous positions of the tool edges tips and change with respect to time (Kaliński [41]). The momentary position of the cutter edge no. *l* is described by the immersion angle *ϕ_l_* = *ϕ_l_*(*t*). It corresponds to the temporary position of CE no. *l*, and the axes *y_l_*_1_, *y_l_*_2_, and *y_l_*_3_ are the coupling axes of this CE (Kaliński [41]). During the machining process, not all edges are cutting the material at any given time. The cutting edges were labelled “active”.

Moreover, the scheme of the milling process shows the following:

- Rake angle *γ*_0_ and clearance angle *α*_0_, as elements of edge geometry in the orthogonal plane;
- Cutting edge angle *κ_r_*;
- Force *F_yl_*_1_, acting in the direction of the nominal cutting speed *v_c_*;
- The current thickness of the cutting layer *h_l_* and the force acting in its direction—*F_yl_*_2_;
- The current width of the cutting layer *b_l_* and the force acting in its direction—*F_yl_*_3_;
- Depth of cutting *a_p_*;
- Milling diameter *D*;
- Milling widths *B*_1_ and *B*_2_; for full milling, *B*_1_ = *B*_2_; down milling, *B*_1_ < *B*_2_; and up milling, *B*_1_ > *B*_2_;
- Local coordinate system *x_r_*_1_, *x_r_*_2_, and *x_r_*_3_ of the RFE;
- Conventional point S of the contact of the tool with the workpiece (Kaliński [41], Kaliński et al. [45]) and the non-rotating coordinate system *x*_1_, *x*_2_, and *x*_3_ for this point, but moving linearly with respect to the workpiece.

An important goal is to formulate a model of the influence of the changing contact zone of the cutting tool with the workpiece on the dynamic characteristics of the cutting process. Thus, the characteristics “cutting force—zone geometry—cutting velocity” require both theoretical and experimental investigations in the case of intermittent cutting (Ehmann et al. [55], Lee et al. [56]). The above is faced with a number of obstacles, caused by the following:

- Various operating regimes, that is to say, pure ploughing, simultaneous ploughing, and shearing, and shearing may govern the cutting process. Based on linearized cutting dynamics models characterizing these regimes, a generalized approach to dealing with nonlinear force effects is being proposed by Yoon and Ehmann [57];
- The need to determine the constitutive relationship of the processed material. The purpose of the research is to determine the characteristics between normal stress, plastic deformation, deformation rate, and temperature, for various cut materials. Within a range of elasticity, the mechanical properties of the material (i.e., Young modulus and Poisson’s ratio) are well described. Within a range of plasticity, the appropriate relationship becomes more complicated, and thus requires experimental investigation (Kpenyigba et al. [58]);
- The need to evidence the hypothesis about the flexible workpiece plasticity existence in the contact zone of edge and workpiece, and its influence on the cutting process dynamic characteristics (Faure et.al. [59]). New cutting force characteristics can be applied for further application in the simulation model;
- The need to verify cutting force characteristics of the intermittent cutting process dynamics, considering in the calculation model the workpiece nonlinearity in a range of plastic deformations (Castro et al. [60]).

Owing to the above difficulties, the mechanistic modeling of the cutting force is applied to numerically estimate the components of the cutting force (Fei et al. [1], Kiran and Kayacan [61]), which seems to be a more convenient attempt. For the temporary point of contact of the tool edge with the workpiece, modeled as CE no. *l*, a proportional model of the dynamics of the cutting process was adopted (Kaliński and Galewski [40], Kaliński [41]), which also takes into account the effects of internal and external modulation of the layer thickness and the edge exit from the workpiece. This approach is justified by significant (above 100 m/min) cutting speed values (Kaliński [41]). According to the assumptions of the adopted model of the cutting process, and taking into account the changes in the thickness *h_l_*(*t*) and width *b_l_*(*t*) of the cutting layer over time, the components of cutting forces were obtained in the following form (Kaliński et al. [45]):

(1) $$F_{yl1}\left(t\right)=\left\{\begin{array}{cc} k_{dl}b_{l}\left(t\right)h_{l}\left(t\right), & h_{l}\left(t\right)>0\;˄\;b_{l}\left(t\right)>0,\\ 0, & h_{l}\left(t\right)\leq 0\;˅\;b_{l}\left(t\right)\leq 0, \end{array}\right.$$

(2) $$F_{yl2}\left(t\right)=\left\{\begin{array}{cc} \mu_{l2}k_{dl}b_{l}\left(t\right)h_{l}\left(t\right), & h_{l}\left(t\right)>0\;˄\;b_{l}\left(t\right)>0,\\ 0, & h_{l}\left(t\right)\leq 0\;˅\;b_{l}\left(t\right)\leq 0, \end{array}\right.$$

(3) $$F_{yl3}\left(t\right)=\left\{\begin{array}{cc} \mu_{l3}k_{dl}b_{l}\left(t\right)h_{l}\left(t\right), & h_{l}\left(t\right)>0\;˄\;b_{l}\left(t\right)>0,\\ 0, & h_{l}\left(t\right)\leq 0\;˅\;b_{l}\left(t\right)\leq 0, \end{array}\right.$$

where

$b_{l}\left(t\right)=b_{D}-\Delta b_{l}\left(t\right)$,

$h_{l}\left(t\right)=h_{Dl}\left(t\right)-\Delta h_{l}\left(t\right)+\Delta h_{l}\left(t-\tau_{l}\right)$,

*b_D_*—desired cutting layer width; *b_D_* = *a_p_*/sin *κ_r_* (Mazur et al. [50]);

Δ*b_l_*(*t*) — dynamic change in cutting layer width for CE no. *l*;

*h_Dl_*(*t*)—desired cutting layer thickness for CE no. *l*; *h_Dl_*(*t*) ≅ *f_z_* sin *κ_r_* cos*ϕ_l_*(*t*) (Mazur et al. [50]);

Δ*h_l_*(.)—dynamic change in cutting layer thickness for CE no. *l*;

*k_dl_*—average dynamic specific cutting pressure for CE no. *l*;

*μ_l_*_2_, *μ_l_*_3_—cutting force ratios for CE no. *l*, as quotients of forces *F_yl_*_2_ and *F_yl_*_1_, and forces *F_yl_*_3_ and *F_yl_*_1_;

*τ_l_ –* time-delay between the same position of CE no. *l* and of CE no. *l*–1;

*κ_r_*—cutting edge angle;

*f_z_*—feed per tooth; *f_z_* = *v_f_*/(*nz*);

*z*—number of milling cutter teeth.

It is worth noting that, in order to explicitly define these forces, it is necessary and sufficient to know only three parameters, *k_dl_*, *μ_l_*_2_, and *μ_l_*_3_ of abstractive significance, the numerical values of which can be adjusted by comparing the respective root mean square (RMS) values of the computational model and the milling process being carried out (see Section 3).

The description of cutting forces for CE no. *l* in six-dimensional space is disclosed and takes the following form (Kaliński et al. [45], Mazur et al. [50]):

(4) $$F_{l}\left(t\right)=F_{l}^{0}\left(t\right)-D_{Pl}\left(t\right)\Delta w_{l}\left(t\right)+D_{Ol}\left(t\right)\Delta w_{l}\left(t-\tau_{l}\right)$$

where

(5) $$F_{l}\left(t\right)=col\left(F_{yl1}\left(t\right),F_{yl2}\left(t\right),F_{yl3}\left(t\right),0,0,0\right),$$

(6) $$F_{l}^{0}\left(t\right)=col\left(k_{dl}b_{D}h_{Dl}\left(t\right),\mu_{l2}k_{dl}b_{D}h_{Dl}\left(t\right),\mu_{l3}k_{dl}b_{D}h_{Dl}\left(t\right),\;0,\;0,\;0\right),$$

(7) $$D_{Pl}\left(t\right)=\left[\begin{array}{cc} \begin{array}{ccc} 0 & k_{dl}\left(b_{D}-\Delta b_{l}\left(t\right)\right) & k_{dl}h_{Dl}\left(t\right)\\ 0 & \mu_{l2}k_{dl}\left(b_{D}-\Delta b_{l}\left(t\right)\right) & \mu_{l2}k_{dl}h_{Dl}\left(t\right)\\ 0 & \mu_{l3}k_{dl}\left(b_{D}-\Delta b_{l}\left(t\right)\right) & \mu_{l3}k_{dl}h_{Dl}\left(t\right) \end{array} & 0_{3\times 3}\\ 0_{3\times 3} & 0_{3\times 3} \end{array}\right],$$

(8) $$D_{Ol}\left(t\right)=\left[\begin{array}{cc} \begin{array}{ccc} 0 & k_{dl}\left(b_{D}-\Delta b_{l}\left(t\right)\right) & 0\\ 0 & \mu_{l2}k_{dl}\left(b_{D}-\Delta b_{l}\left(t\right)\right) & 0\\ 0 & \mu_{l3}k_{dl}\left(b_{D}-\Delta b_{l}\left(t\right)\right) & 0 \end{array} & 0_{3\times 3}\\ 0_{3\times 3} & 0_{3\times 3} \end{array}\right],$$

(9) $$\Delta w_{l}\left(t\right)=col\left(q_{zl}\left(t\right),\Delta h_{l}\left(t\right),\Delta b_{l}\left(t\right),0,\;0,\;0\right),$$

(10) $$\Delta w_{l}\left(t-\tau_{l}\right)=col\left(q_{zl}\left(t-\tau_{l}\right),\Delta h_{l}\left(t-\tau_{l}\right),\Delta b_{l}\left(t-\tau_{l}\right),0,\;0,\;0\right),$$

where $q_{zl}\left(t\right)$—relative displacement of edge tip and workpiece along direction *y_l_*_1_ at instant of time *t* and $q_{zl}\left(t-\tau_{l}\right)$—relative displacement of edge tip and workpiece along direction *y_l_*_1_ at instant of time *t* −*τ_l_*.

The illustrated considerations take into account all the most important non-linear effects observed in real milling operations, that is to say (Kaliński et al. [45]):

- The loss of contact between the cutting tool edge and the workpiece, owing to the lower limitation of the cutting force characteristics (1)–(3);
- The geometric non-linearity resulting from the dependence on the dynamic change in the width of the cutting layer (see Equations (7) and (8)).

As a result of modeling the dynamics of the milling process, a hybrid system is obtained, which consists of the following (Figure 1):

- A modal subsystem, i.e., a stationary model of a flexible workpiece moving with a given feed speed *v_f_*. Its behavior is described by the vector of its modal coordinates **a**. No FEM is included, and the parameters of the modal model, unlike in Kaliński et al. [45], are obtained only from the experiment. Therefore, taking into account the finite number of normal modes *mod* of the subsystem, we define its dynamic properties with the following:
  **Ω** = *diag*(*ω*_0*i*_)—matrix of angular natural frequencies of the modal subsystem; *i* = 1, …, *mod*; its square is also called the *stiffness modal matrix*;
  $\Psi =\left[\begin{array}{ccc} \Psi_{1} & \ldots & \Psi_{mod} \end{array}\right]$—matrix of the considered mass-normalized normal modes of the modal subsystem; *i* = 1, …, *mod*;
  **Ζ** = *diag*(*ζ*_*i*_)—matrix of dimensionless damping coefficients (also called *modal damping*) of the modal subsystem; *i* = 1, …, *mod*.
  Another important argument for the desirability of description in modal coordinates is that the physical properties of the workpiece do not change with time. In the case of processing objects of large dimensions, we deal with a small (approximately 1 per mille) allowance for material removal in relation to the total mass of the object;
- A structural subsystem, i.e., a discrete model (the RFE and the SDE) of a rotating milling cutter with a given spindle speed *n*. The behavior of the subsystem is described by the vector of its generalized coordinates **q**. The small number (i.e., 6) of these coordinates, additionally having an unambiguous physical interpretation, does not justify the desirability of describing the behavior of the dynamics of the rotating tool in modal coordinates (Eksioglu et al. [62]). The dynamic properties of the structural subsystem are defined by inertia **M**, damping **L**, and stiffness **K** matrices. The way of determining these matrices is shown in the Appendix A;
- An abstractive connecting subsystem such as, unlike in Kaliński et al. [45], a set of conventional points of contact of the tool edges with the workpiece. Their generalized coordinates are related to other coordinates through time-dependent constraint equations (Kaliński [41]). This allows to eliminate these generalized coordinates from the description of the behavior of the hybrid system.

### 2.2. Dynamics of the Milling Process of Flexible Workpieces in Hybrid Coordinates

Let us describe the dynamics of the non-stationary hybrid model of the milling process in hybrid coordinates. Then, the matrix equation of the dynamics will take the following form (Kaliński [41], Kaliński et al. [45]):

(11) $$\left[\begin{array}{cc} M & 0\\ 0 & I \end{array}\right]\ddot{\xi }+\left[\begin{array}{cc} L & 0\\ 0 & 2Z\Omega \end{array}\right]\dot{\xi }+\left[\begin{array}{cc} K+\sum_{l=1}^{i_{l}}T_{l}^{T}\left(t\right)D_{Pl}\left(t\right)T_{l}\left(t\right) & -\sum_{l=1}^{i_{l}}T_{l}^{T}\left(t\right)D_{Pl}\left(t\right)W_{l}\left(t\right)\\ -\sum_{l=1}^{i_{l}}W_{l}^{T}\left(t\right)D_{Pl}\left(t\right)T_{l}\left(t\right) & \Omega^{2}+\sum_{l=1}^{i_{l}}W_{l}^{T}\left(t\right)D_{Pl}\left(t\right)W_{l}\left(t\right) \end{array}\right]\xi =\;=\left[\begin{array}{c} \sum_{l=1}^{i_{l}}T_{l}^{T}\left(t\right)F_{l}^{0}\left(t\right)+T_{l}^{T}\left(t\right)D_{Ol}\left(t\right)\Delta w\left(t-\tau_{l}\right)\\ -\sum_{l=1}^{i_{l}}W_{l}^{T}\left(t\right)F_{l}^{0}\left(t\right)-W_{l}^{T}\left(t\right)D_{Ol}\left(t\right)\Delta w\left(t-\tau_{l}\right) \end{array}\right]$$

where

$\xi =\left\{\begin{array}{c} q\\ a \end{array}\right\}\;$—vector of hybrid coordinates of the hybrid system;

**T***_l_*(*t*)—transformation matrix of displacements’ vector **q** from the *x_r_*_1_, *x_r_*_2_, and *x_r_*_3_ coordinates of the RFE, to the coordinate system *y_l_*_1_, *y_l_*_2_, and *y_l_*_3_ of CE no. *l* (Kaliński [41]) (see the Appendix A);

**W***_l_*(*t*)—transformation matrix between the displacement vector in modal coordinates **a**, and displacements in the coordinate system *y_l_*_1_, *y_l_*_2_, and *y_l_*_3_ of CE no. *l* (Kaliński [41]);

*i_l_*—number of “active” CEs.

The structural subsystem and the modal subsystem remain stationary before the connection. Only after they are combined into a hybrid model does the entire system become non-stationary, owing to the apparent variability in time resulting from the given movements of the subsystems. Time domain simulations based on Equation (11) take into account the change in the geometric positions of the instantaneous contacts of the tool edges with the workpiece.

In order to identify the modal model of the flexible workpiece (being part of Equation (11)), the matrix of normal modes **Ψ**, the matrix of dimensionless damping coefficients **Z**, and the matrix of corresponding angular natural frequencies **Ω** of the modal subsystem should be determined. Thanks to separation of the modal subsystem from the entire non-stationary structure, a significant reduction in the size of the model to only a few modes is obtained. The number of modes depends on their importance and the need for selecting modes for further analysis.

Normal modes remain unchanged over time owing to the immobility of the modal subsystem during the machining process. Thanks to this, only the normal modes **Ψ**, dimensionless damping coefficients **Z**, and angular frequencies **Ω** can be identified, unlike in [45], by the methods of experimental modal analysis (EMA) on the workpiece installed on the milling machine table.

In various zones of the milled surface, different dominant vibration modes can be observed, especially in the case of large workpieces or workpieces with complicated geometrical shapes. Therefore, in the case of the modal model obtained directly from the experiment, it is assumed that the dominant modes are those identified on the basis of the frequency response function (FRF), measured only at points lying on the machined surface.

## 3. Experimentally Aided Operational Virtual Prototyping

**Figure 2:**
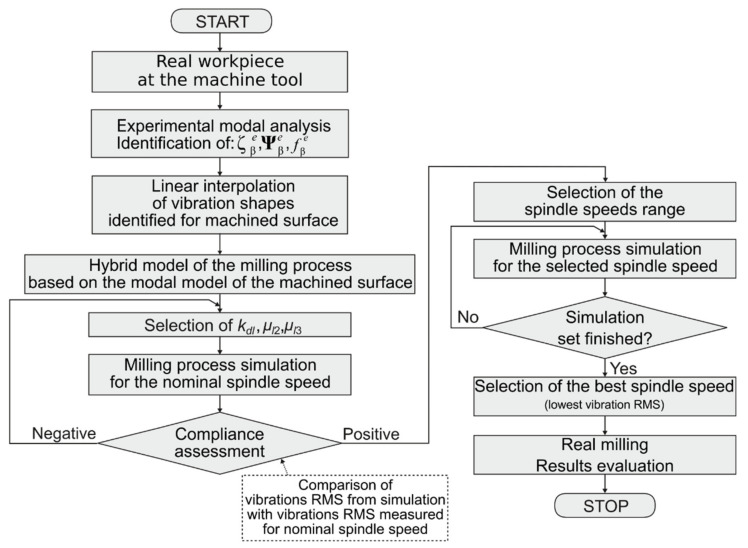
Scheme of the EAOVP on the basis of linear interpolation.

In Kaliński et al. [45], the technique of experimentally aided virtual prototyping (EAVP), based on the validated modal approach, was elaborated. As a result of progressive modifications introduced in this method, a new technique of experimentally aided operational virtual prototyping (EAOVP) was proposed, based solely on the experimental modal model of the workpiece, linear interpolation of the identified modes, and simulation of the milling process performance. The latter resulted in the following spindle speed selection procedure (Figure 2):

1. Estimation of modal parameters using only EMA. The latter concerns natural frequencies $f_{\beta }^{e}$, modal damping coefficients $\zeta_{\beta }^{e},$ and normal modes $\Psi_{\beta }^{e}$, =1,…, mod. Only excitation and response measurement points lying on the machined surfaces are taken into consideration.
2. Linear interpolation of the identified modes along the toolpath on the workpiece surface modelled in the simplified manner.
3. Dominant modes are selected in order to be used in the modal subsystem of the hybrid model of the milling process.
4. Determination of simulation parameters (*k_dl_*, *μ_l_*_2_, *μ_l_*_3_) of the cutting process, adjusting the results of milling simulations carried out for the nominal (standard) spindle speed to the results of the real cutting performed for the same spindle speed.
5. Milling process simulations for various spindle speeds in the selected range. Cutting process parameters specified in p. 4 are applied.
6. For a set of simulated milling processes, vibration levels are observed, i.e., the root mean square (RMS) of relative tool–workpiece displacements; based on these results, the best spindle speed is selected.
7. Performance of the real milling process with the selected best spindle speed.

The advantage of the proposed method over the EAVP (Kaliński et al. [45]) is as follows:

- The precise obtaining of the best value of the spindle speed requires a much shorter time. After all, the calculation model of the workpiece is simplified only to the milling area model. There is no need to prepare a complex finite element model (FEM) of the whole workpiece and to correlate it according to the modal test, which is a very time-consuming task;
- Only modes that are important from the point of view of modelling the behavior of the machined surface are considered;
- Dominant frequencies and modal damping coefficients are identified directly from modal tests, and thus they are more accurate;
- Selection of dominant modes is natural as only important modes are observed when the workpiece excitation is applied on the machined surface. Because of this, the number of normal modes is usually lower than in the case of the previous technique of EAVP (Kaliński et al. [45]).

The presented algorithm (Figure 2) does not require the purchase of expensive commercial FEM software, as well as the involvement of expensive and complex systems of measurement and control equipment. The proprietary linear interpolation procedures and simulation of the hybrid model of the milling process were launched with the use of free programming environments. Hence, the algorithm in the disclosed version can easily be directly implemented in an engineering practice where the “real milling” schema block will be implemented without “results evaluation”.

## 4. Simulation and Experimental Results

### 4.1. The Experimental Setup

**Figure 3:**
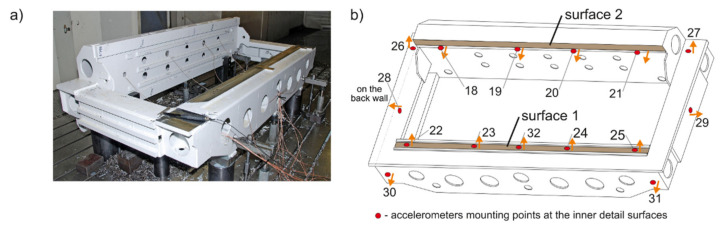
Test workpiece: (**a**) clamped on the machine table and (**b**) simplified scheme of the workpiece with indicated mounting points for accelerometers [45].

The experimental research was performed on a large workpiece selected from the common production program of a cooperating industrial company (PHS Hydrotor S.A., Tuchola, Poland), because this is the only way to effectively solve large-size machining problems (Uriarte et al. [43]). The workpiece was made of STW22 03M steel and had a total size of 2061 × 1116 × 540 mm (Figure 3a). Modal tests as well as the succeeding milling operations were performed on the workpiece clamped on a table of the MIKROMAT 20V (VEB Mikromat, Dresden, Germany) portal machining center. An appropriate system of measuring equipment and signal processing was used in the research (Kaliński et al. [45]).

Two surfaces of the workpiece were milled (Figure 3b). For surface 1, two passes were performed as one complete operation. In the first pass, i.e., full face milling, the tool (Sandvik R390-044C4-11M060, Sandvik AB, Stockholm, Sweden) moved from the left (i.e., near accelerometer no. 22) to the right. In the second pass, i.e., down milling, the tool moved in the opposite direction (i.e., starting from near accelerometer 25). For surface 2, only one down milling pass was made, with the tool (Sandvik R390-125Q40-17H, Sandvik AB, Stockholm, Sweden) moving from left to right (i.e., starting around accelerometer 18). The primary guideline for selecting measurement points’ positions was to enable measuring vibration signals along the milled surfaces. Thus, five DJB A/120/V ±75g (DJB Instruments, Suffolk, UK) accelerometers were placed at equal intervals under the first milled surface, and four DJB A/120/V accelerometers under the second one (Figure 3b).

The standard parameters (Kaliński et al. [45]) used in the milling process are derived from the common production scheme of the cooperating industry partner (Table 1). The use of tools with cutting edge angles *κ* = 90° means that taking into account the bending phenomenon of the tool system, typical for relatively large cutter diameters, is not required in the calculation model (Totis et al. [44]). The vibration level observed during milling performed with these parameters was recognized as a reference to the evaluation of the results of the proposed method of searching for the best spindle speed.

### 4.2. Experimental Modal Identification

Experimental modal studies of the workpiece were performed for each mounting point of the accelerometers (see Figure 3b) with the use of a PCB 086C03 (PCB Piezotronics Inc. Depew, NY, USA) modal hammer. Using the polyreference-least squares complex frequency domain (p-LSCFD) method (Kaliński [41], Heylen et al. [63]) applied to force-acceleration FRFs, *mod* = 7 dominant natural frequencies and modal damping coefficients for every surface were identified (Table 2). This means that the experimental modal models were correctly determined in the range of frequencies up to 500 Hz.

For points located on the machined surface, the modes were scaled to unit modal masses, so that the **Ψ** matrix of normal modes of the modal subsystem was mass-normalized.

Necessary normal modes of a modal subsystem were computed from complex modes by calculating their amplitudes. Only modes having well stabilized poles in the stability diagram and the low phase deviation (MPC (modal phase collinearity) > 0.85) (Kaliński [41], Maia and Silva [64]) were selected. During identification, only sensors located on the selected surface were considered. All available inputs were utilized, i.e., excitations of every sensor located on the surface. The results obtained for sensors located at a further distance from the excitation place were not taken into consideration during identification. It must be also noted that excitation was applied near every sensor, so that the signal to noise ratio was high. In a given condition, the p-LSCFD method produced reliable results. An important advantage of the p-LSCFD is generally good identification of highly damped poles (Heylen et al. [63]), which was the case for the experimental workpiece used during the impulse tests. The applied modal test and identification procedure focus on the vibration modes important for the milling zone. It naturally eliminates modes that should weekly influence the machined surface dynamics. The selection of sensors placed on the machined surface and excitations perpendicular to this surface resulted in obtaining a small number of normal modes, but ones important from the point of view of reliable milling dynamics description.

### 4.3. Linear Interpolation

**Figure 4:**
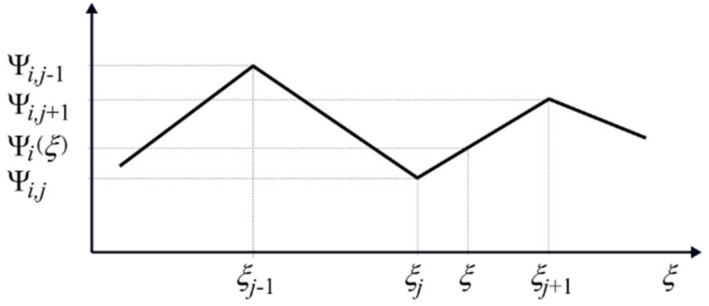
Linear interpolation of the normal mode vector value $\Psi_{i}\left(\xi \right)$ for the coordinate *ξ* between measuring points *j* and *j* + 1.

The number of sensors *j_s_* was limited, so these modes are defined for a few points only, not dense enough for the purpose of milling simulations. In order to solve this problem, an original technique was developed that, for given geometrical data of a machined surface, computes displacements for scaled modes identified from modal tests and interpolates them for other surface points. For this purpose, the use of linear interpolation was proposed. The scheme of such linear interpolation of the identified normal mode vector $\Psi_{i}=col\left(\Psi_{i1},\;\ldots ,\;\Psi_{ij},\;\ldots ,\Psi_{ij_{s}}\right)$, *i* = 1 …, *mod*, for the coordinate value *ξ* lying between the measuring points *j* and *j* + 1 corresponding to the sensors’ positions (Figure 4) shows that the appropriate interpolated value has the following form:

(12) $$\Psi_{i}\left(\xi \right)=\Psi_{ij}+\frac{\xi_{\left(t\right)}-\xi_{j}}{\xi_{j+1}-\xi_{j}}\left(\Psi_{i,j+1}-\Psi_{ij}\right).$$

The values of the vectors of normal modes $\Psi_{i}\left(\xi \right)$, *i* = 1, …, *mod*, thus interpolated, can be used to create the matrix of transformation $W_{l}\left(t\right)$ (compare Formula (11)) between the displacement vector in modal coordinates **a** and the generalized displacement vector in coordinate system *y_l_*_1_, *y_l_*_2_, and *y_l_*_3_ of CE no. *l*. It should be noted that, for changing the position of the contact between the edge top of the tool and the workpiece, where $\xi =\xi \left(t\right)$, we obtain the following:

(13) $$W_{l}\left(t\right)=W_{l}\left(t,\xi \left(t\right)\right)=\left[\begin{array}{cc} \Theta_{l}{\left(t\right)}_{3\times 3} & 0_{3\times 3}\\ 0_{3\times 3} & \Theta_{l}{\left(t\right)}_{3\times 3} \end{array}\right]C_{W}\hat{\Psi }\left(\xi \left(t\right)\right),$$

where

$\Theta_{l}\left(t\right)$—matrix of directional cosines between the axes *y_l_*_1_, *y_l_*_2_, and *y_l_*_3_ of CE no. *l*, and the axes of the coordinate system *x*_1_, *x*_2_, and *x*_3_ (Figure 1);

$C_{W}=col\left(0,\;0,\;1,\;0,\;0,\;0\right)$;

$\hat{\Psi }\left(\xi \left(t\right)\right)={\left[\begin{array}{c} \Psi_{1j}+\frac{\xi \left(t\right)-\xi_{j}}{\xi_{j+1}-\xi_{j}}\left(\Psi_{1,j+1}-\Psi_{1j}\right)\\ \vdots \\ \begin{array}{c} \Psi_{ij}+\frac{\xi \left(t\right)-\xi_{j}}{\xi_{j+1}-\xi_{j}}\left(\Psi_{i,j+1}-\Psi_{ij}\right)\\ \begin{array}{c} \vdots \\ \Psi_{mod,j}+\frac{\xi \left(t\right)-\xi_{j}}{\xi_{j+1}-\xi_{j}}\left(\Psi_{mod,j+1}-\Psi_{mod,j}\right) \end{array} \end{array} \end{array}\right]}^{T}$.

After interpolation or extrapolation (for surface points of the workpiece lying outside sensors’ positions), it is possible to obtain these values for a set of points on the machined surface much larger than the set of sensor positions. The observed normal modes were of a low degree, so a mode aliasing was avoided.

The approximate time to obtain the expected modal model for one surface of the workpiece using a laptop computer equipped with an Intel Core i7-6700HQ 2.60 GHz CPU and 32 GB RAM was about 15 min, which is much shorter than the 240 min needed in the case of assuring compatibility between the modal experimental results and the FEM (Kaliński et al. [45]). The interpolated mass-normalized normal modes for points lying on the machined surface were then exported to the original AMIKRO4 milling process simulation software. Next, the simulations using the modal model based on only seven dominant modes were performed for a set of spindle speeds.

### 4.4. Spindle Speed Selection

According to the approach of searching for the best spindle speed using EAOVP during milling high-sized workpieces (Figure 2), the next phase of this procedure involves simulations of the face milling process of the machined surfaces performed for standard parameters. These simulations utilize a non-stationary calculation model and are performed in order to obtain the compliance of the RMS values of simulated vibrations’ displacements with the values measured in standard production conditions. Other milling process model simulations’ validation quantities may be used as well. However, the simulated models are non-stationary and strongly non-linear, so the measures usually utilized for steady state vibrations are not applicable here. The concept based on RMS seems to be the best measure of displacement assessment in the studied cases (Kaliński et al. [45]).

One can observe the adjusted parameters *k_dl_*, *μ_l_*_2_, and *μ_l_*_3_ of the hybrid model used during milling simulation for the nominal spindle speed (i.e., standard milling) in order to satisfy the condition of compliance of the RMS values obtained from machining and the corresponding RMS values of simulated plots (Table 3). In the adopted mechanistic model of cutting forces, the meaning of the above-mentioned coefficients is abstract. Therefore, there is neither an analytical nor an experimental method that would unambiguously allow their value to be determined. All that remains is to estimate them. Owing to the similarity of the analyzed milling operations of a large-size object, the initial values were identical to those considered in Kaliński et al. [45]. After adjusting them, these values differed slightly from the initially adopted ones.

Milling simulations were performed for a specific spindle speed range. Spindle speeds below the lower limits of the adopted ranges were omitted, because the selection of the best spindle speed was to result not only in a reduction of the vibration level, but also in an increase in the efficiency of the milling process.

For each simulated spindle speed, the vibration level was observed and three indicators were calculated, which are presented in the appropriate figures. These are as follows (Kaliński et al. [45]): *RMS*_95%_, i.e., RMS of relative tool-workpiece displacements calculated for 95% of the entire cutting time; *A_max_*, i.e., the maximum amplitude of relative tool–workpiece displacements calculated for the same period as for RMS_95%_; and *RMS_95% MR_*, i.e., RMS of relative tool–workpiece displacements calculated for 95% of the whole total cutting time, but in relation to the average value of the considered vibrations (*MR* –related to the average). The latter index (analogous to the standard deviation of vibration) can be interpreted as an indicator of the vibration level relative to the static displacement of the workpiece surface caused by the action of the tool. This corresponds best to the piezoelectric accelerometer method of measuring vibration during the actual milling process, when low frequency vibrations and static deflections are ignored.

**Figure 5:**
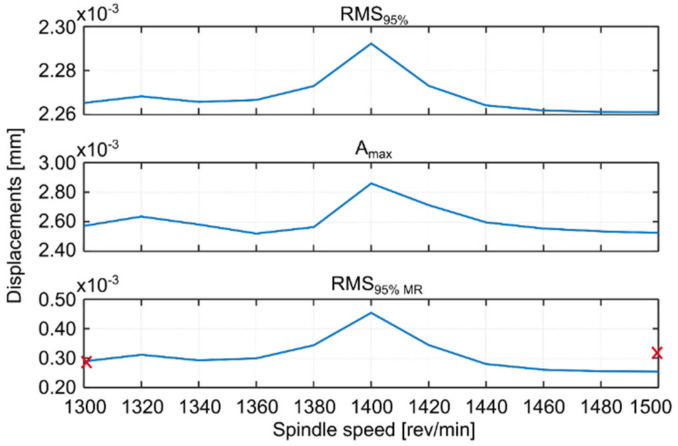
EAOVP for full milling of the workpiece, surface 1, indices values with respect to the simulated spindle speed, x—average results of measurements.

**Figure 6:**
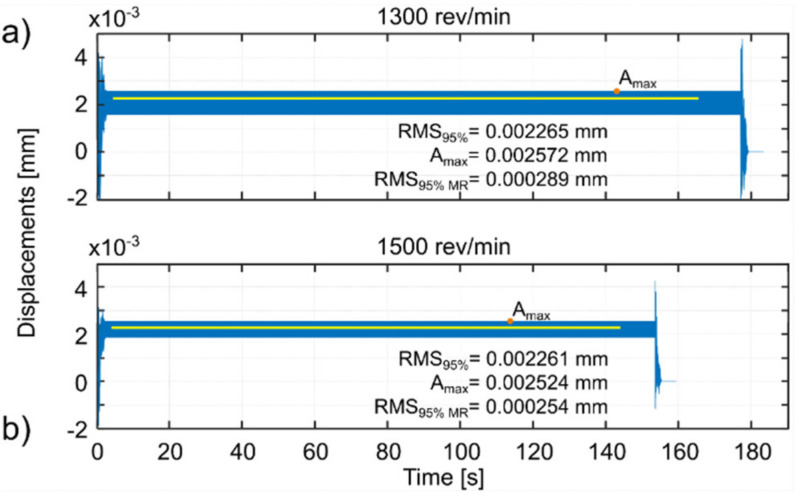
EAOVP, simulation results for full milling of surface 1 at (**a**) the standard spindle speed of 1300 rev/min and (**b**) the best spindle speed of 1500 rev/min.

**Figure 7:**
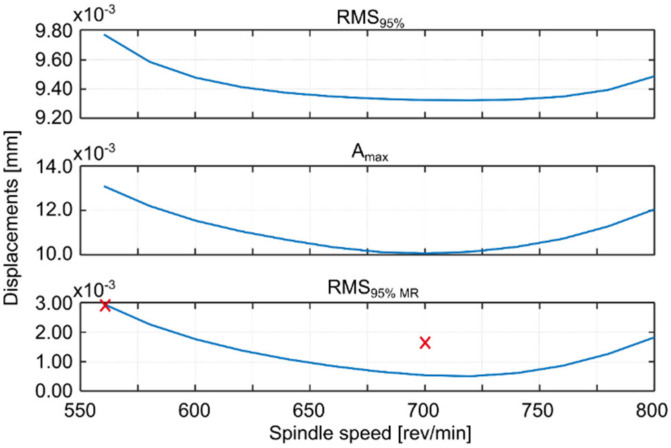
EAOVP for down milling of the workpiece, surface 2, indices values with respect to the simulated spindle speed, x—average results of measurements.

**Figure 8:**
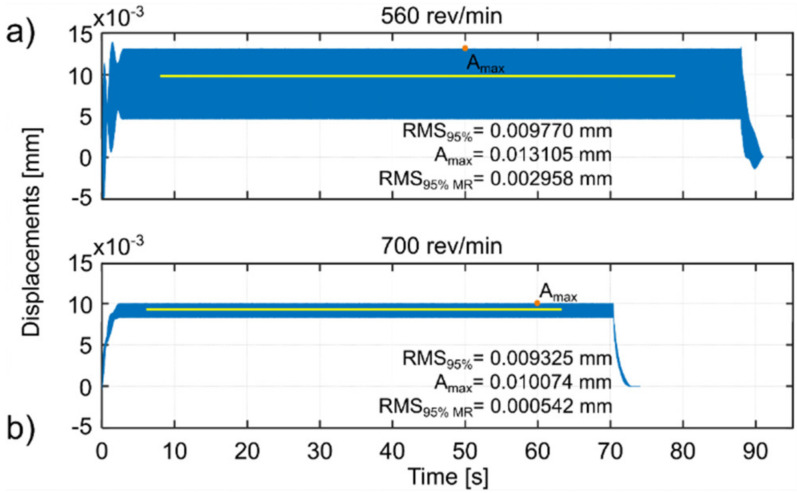
EAOVP, simulation results for down milling of surface 2 at (**a**) the standard spindle speed of 560 rev/min and (**b**) the best spindle speed of 700 rev/min.

Based on the simulations performed, the best spindle speed was selected for surface 1 (Figure 5) and appropriate plots of the simulation results, at the standard and best spindle speeds, are presented in Figure 6. Similarly the best spindle speed was selected for surface 2 (Figure 7) and relevant plots of the simulation results are presented in Figure 8. The predicted RMS values for the best spindle speeds are shown in Table 3. Subsequently, such best parameters were used for milling processes, whose results are presented and discussed in the next subsection.

The proposed method uses modal test results directly. This saves a lot of time and the requirements for the measuring equipment used can also be lower. For example, there is no need for time-consuming extensive modal tests of the whole workpiece structure and the complicated FEM correlation (Kaliński et al. [45]). Milling simulations are performed for a simplified hybrid model, which are faster than in the case of full FEM of the workpiece and support. When the full FEM is utilized, it is often difficult to predict which modes will be important during milling, so a greater number of them must be taken into account. Meanwhile, frequencies of normal modes identified in a modal test are more accurate than those calculated from FEM, even if the FEM is very well correlated. In the EAOVP, during analysis of the respective machined surface, only data for points lying on the selected surface are taken into consideration. Thanks to this, analyses are mainly performed for high quality signals, ignoring weakly excited modes and measurement points that have low influence on the subjected modes. Moreover, it can be difficult to obtain, on the basis of the latter, promising results on massive objects mounted on a milling machine with the help of numerous supports and clamps.

The proposed method provides a solution that improves the milling process in such a way that the vibration level of the workpiece approaches the minimum (compare Figure 5 and Figure 7).

### 4.5. Real Milling Results

**Figure 9:**
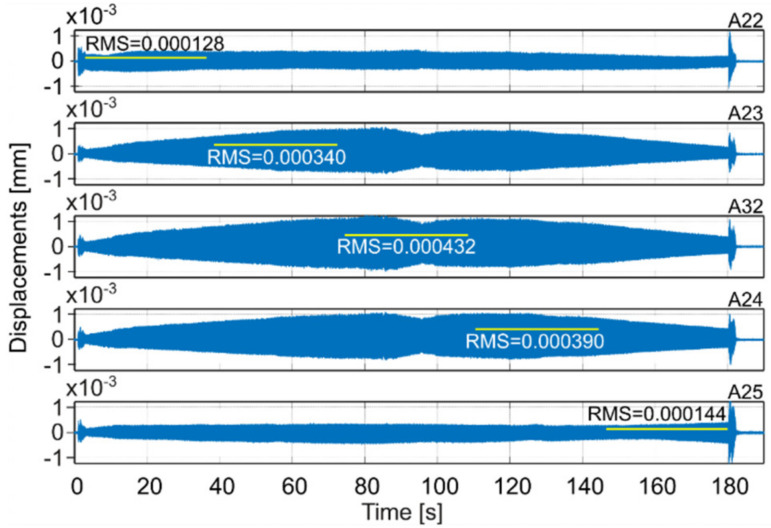
Vibrations of the workpiece during full milling of surface 1 at standard parameters (see Table 4) [45].

**Figure 10:**
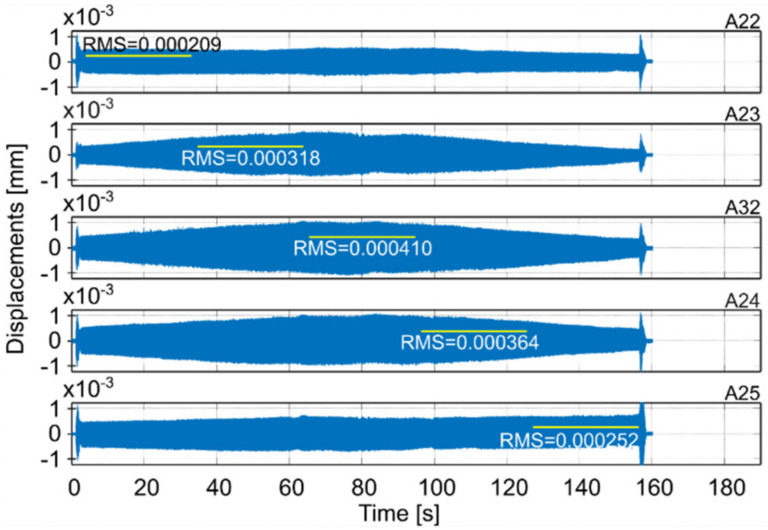
Vibrations of the workpiece during full milling of surface 1 at the best parameters (see Table 4).

**Figure 11:**
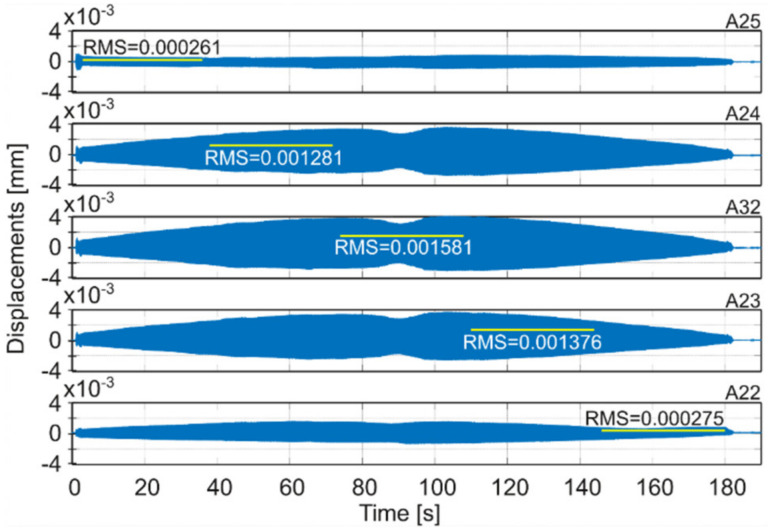
Vibrations of the workpiece during down milling of surface 1 at standard parameters (see Table 4).

**Figure 12:**
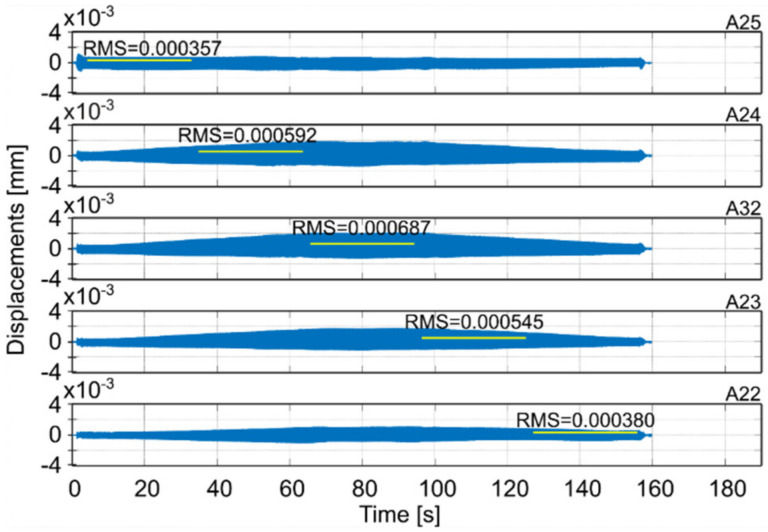
Vibrations of the workpiece during down milling of surface 1 at the best parameters (see Table 4).

**Figure 13:**
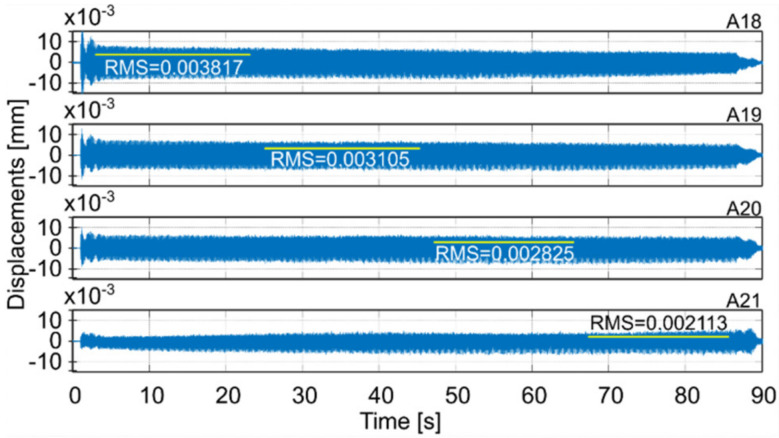
Vibrations of the workpiece during down milling of surface 2 at standard parameters (see Table 4).

**Figure 14:**
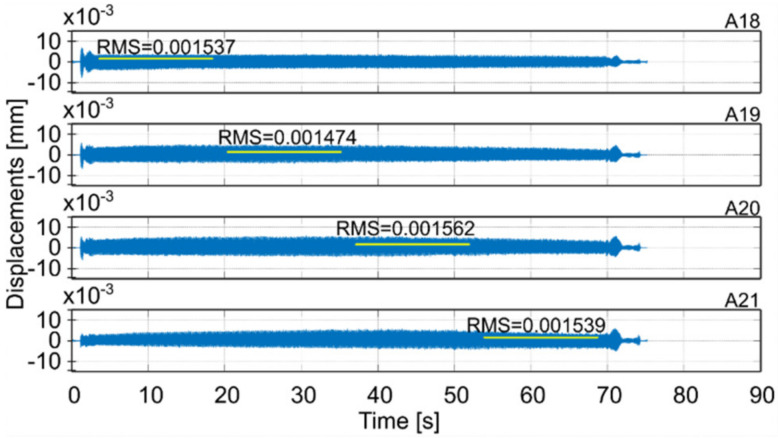
Vibrations of the workpiece during down milling of surface 2 at the best parameters (see Table 4).

Milling operations were performed for both surfaces of the workpiece, with spindle speeds selected according to standard parameters and the method presented in this paper. Table 4 presents the selected milling parameters, wherein the selection of the best speed corresponds to that obtained from simulations based on the EAOVP. In Table 5 and Table 6, the sign “Axx” represents the number of the indicated accelerometer (see Figure 3b). In turn, Table 5 shows the RMS values of displacements for the milling operations performed, observed at the measurement points while moving the tool over the surroundings of a given accelerometer. The displacements values in Figure 9, Figure 10, Figure 11, Figure 12, Figure 13 and Figure 14 are presented as the results of double integration of the measured accelerations (during integration, the signal was filtered with an ideal high-pass filter with a cutoff frequency of 20 Hz). Table 6 presents the same data, but as relative values, to help note the better results provided towards vibration suppression by the proposed approach, wherein the vibration reduction is marked with a “-“ sign.

As mentioned earlier, the milling results with standard parameters were treated as a reference for further tests. The presented method for selecting the best spindle speed has been successfully tested.

The use of the EAOVP to milling surfaces 1 and 2 leads to a slight underestimation of all the predicted results in relation to the measured average vibration displacements, in each milling case (Figure 5 and Figure 7, Table 5). However, the above is irrelevant because the most important thing is that this approach gave better milling results than with the standard types and, moreover, in a significantly shorter time.

Compared with the standard technology (Table 6), in the case of milling surface 1, a significant reduction in the vibration level (RMS displacement) was achieved in the down milling type (by 46.4%), and in the case of full milling, a slight increase in RMS vibrations by 8.3%. The above was accompanied by an increase in the spindle speed by 15% (Table 4), which made it possible to shorten the main time of the removal of the allowance (two passes) by 0.79 min, i.e., by 13%. In the case of milling surface 2, an increase in spindle speed of 25% was achieved (Table 4), with a significant reduction in RMS vibrations of 48.5% (Table 6), which resulted in a reduction in the main time of the pass of 0.29 min, i.e., by 20%.

Table 6 also presents a comparison of the relative changes in the RMS value during milling operations obtained with the EAOVP method proposed in the current article and the EAVP method described earlier by Kaliński et al. [45]. For full milling of surface 1, the average RMS value increased slightly, although not significantly. On the other hand, during down milling, the reduction in the average RMS value is comparable in both methods. Further, in the case of milling surface 2, it is even more effective. The above proves that the required accuracy of surface treatment is actually met.

### 4.6. Assessment of the Profitability of the EAOVP

In order to estimate the profitability resulting from the implementation of the developed innovative solution, the relevant production standards of technological times were compared (Feld [65]). So, let us analyze the reconsidered process of face milling of the large workpiece surfaces on the MIKROMAT 20V portal machining center, where two passes are performed per surface 1, i.e., full milling and down milling, and one pass per surface 2 (see Section 4.1). According to the standard technology, such processing has so far been performed with a time per unit (approximately equal to the operation time), which, for three passes, is as follows:

*t_j_* = 2 × (*l_pk_*_1,2_ × *t_pk_*_1,2_ + *t_g_*_1,2_) + *l_pk_*_3_ × *t_pk_*_3_ + *t_g_*_3_ (14)

where

- *l_pk_*_1,2_—number of inspection cuts for pass 1 and 2;
- *t_pk_*_1,2_—time of inspection cut of pass 1 and 2;
- *t_g_*_1,2_—main time of pass 1 and 2;
- *l_pk_*_3_—number of inspection cuts for pass 3;
- *t_pk_*_3_—time of inspection cut of pass 3;
- *t_g_*_3_—main time of pass 3.

For the sake of simplicity, other components of the auxiliary time *t_p_* were omitted in Formula (14). Assuming *l_pk_*_1,2_ = *l_pk_*_3_ = 1, *t_pk_*_1,2_ = *t_g_*_1,2_ = 2.96 min, *t_pk_*_3_ = *t_g_*_3_ = 1.44 min, we get *t_j_* = 14.72 min.

In the implementation of the proposed innovative solution, the main time of pass 1 and 2 is *t_g_*_1,2_ = 2.57 min, that of pass 3—*t_g_*_3_ = 1.15 min, and there are no inspection cuts (*l_pk_*_1,2_ = *l_pk_*_3_ = 0). This gives the total unit time of three passes *t_j_* = 6.29 min, which is 57% shorter.

The total time for machining *w* workpieces, with three passes per unit, is thus as follows:

- in the case of standard technology—14.72 × *w* min;
- in the case of the proposed innovative technology—6.29 × *w* min.

The processing time in the case of the proposed innovative technology should be increased by the time of experimental modal analysis (EMA) and linear interpolation for each surface of only the first workpiece, i.e., 2 × approximately 15 min, and the time of two parallel simulation series for each surface, in order to determine the parameters of the hybrid model and the best spindle speed, i.e., approximately 23 min.

The required number of workpieces, *w*, satisfying the condition of profitability in terms of machining time, results from the following inequality:

(15) 14.72w≥6.29w+2×15+23,

which gives w≥6.29. Thus, the minimum time-effective number of workpieces to be made is *w* = 7.

However, in the implementation of the former solution by the EAVP method (Kaliński et al. [45]), the main time of pass 1 and 2 is *t_g_*_1,2_ = 2.79 min, that of pass 3 is *t_g_*_3_ = 1.09 min, and there are no inspection cuts (*l_pk_*_1,2_ = *l_pk_*_3_ = 0). This gives the total unit time of three passes *t_j_* = 6.67 min, which is 55% shorter.

The total time for machining *w* workpieces, with three passes per unit, is thus as follows:

- in the case of standard technology—14.72 × *w* min;
- in the case of the EAVP method—6.67 × *w* min.

The processing time in the case of the EAVP method should be increased by the time of theoretical (i.e., by the finite element method) and experimental modal analysis (EMA) of only the whole first workpiece, i.e., approximately 240 min, and the time of two parallel simulation series for each surface, in order to determine the parameters of the hybrid model and the best spindle speed, i.e., approximately 30 min.

The required number of workpieces, *w*, satisfying the condition of profitability in terms of machining time, results from the following inequality:

(16) 14.72w≥6.67w+240+30,

which gives w≥33.54. Thus, the minimum time-effective number of workpieces to be made is *w* = 34. As the mechanical processing of large-size items in production companies usually concerns small series, the number of which does not exceed 15 items, the proposed EAOVP method is thus more economically viable than the previously proposed EAVP.

The profitability resulting from the implementation of the developed innovative solution, i.e., EAOVP, is thus perceived to be in the category of the following:

- Optimizing the vibration level of the workpiece, leading to improved product quality;
- Determining the minimum time-effective number of workpieces to be made, i.e., *w* = 7;
- Reduction in the cost of the removed material by 50% owing to the lack of inspection cuts, the number of which in the standard technology is equal to the number of working cuts.

## 5. Conclusions

The EAOVP technique based on linear interpolation has proven successful in determining the best spindle speed for face milling of large-size flexible workpieces, in terms of minimizing the level of tool–workpiece vibrations. The results were achieved in less time by eliminating several time-consuming steps. The latter concerns, for example, no need to create FEM and validate modal parameters.

The essence of the proposed method lies in the effective search, within the accepted range of spindle speeds, of the best speed value for which the tool–workpiece vibration level in the milling of the selected surface reaches a minimum. The above does not imply the complete elimination of vibrations (which is impossible anyway), but a significant reduction in their level. The relationship between minimizing the vibration level of the tool–workpiece in large-size machining and the quality of the treated surface has been proven in many scientific elaborations (e.g., Liu et al. [18]). It is also the result of industrial research conducted by the authors (Kaliński et al. [45]). Therefore, the search for a spindle speed value that satisfies the minimization of the vibration level should have a positive impact on the quality of the milled surface.

The full milling type is much better dynamically conditioned, as evidenced by the RMS value of the vibration level at both the standard and the best spindle speed. Hence, the effectiveness of the surveillance, measured by RMS values in both cases, is not that noticeable. On the other hand, in the case of down milling, much less conditioned and much higher RMS values are observed, especially during milling surface 2. Hence, a much better improvement in these values proves the usefulness of the method proposed in the article.

The advantage of EAOVP is a much shorter time to obtain the best spindle speed than in the case of the classic EAVP (Kaliński et al. [45]), which makes it more suitable for use in the practice of production enterprises. The absence of a full FEM in the approach saves time both in building the model and in adapting it to the results of experimental modal tests. With EAOVP, modal tests are also easier and take less time, and you can use fewer measurement points. After all, the tests are limited only to the surfaces to be machined. The assessment of the profitability of implementing the proposed innovative approach should be considered in the category of optimizing the vibration level of the workpiece, resulting in an improvement in the quality of workmanship, as well as a significant reduction in the production standard of the unit execution time. In addition, the reduction in material costs to be removed cannot be overestimated owing to the absence of inspection cuts, a significant number of which actually exist in standard technology.

The selection of the best technological parameters of the machining process through experimental material tests when milling large-size workpieces is time-consuming, expensive, and ineffective. The proposed EAOVP technique meant that the basis for the selection of the best spindle speed was the results of a computer simulation of the computational model, the parameters of which were identified only by the experimental modal analysis (EMA). The number of material machining experiments was limited to two for each surface, i.e., the standard (matching the parameter values to the results of the cutting process simulation) and the best (confirming the effectiveness of the computer prediction).

## Figures and Tables

**Table 1:** Data used for the standard milling process of the test workpiece.

Milled Surface	Length [mm]	Type of Milling	*B*_1_ [mm]	*B*_2_ [mm]	Standard Parameters			Milling Cutter			
					*n* [rpm]	*v_f_* [mm/min]	*a_p_* [mm]	Type	*z*	*D* [mm]	*κ* [°]
1	1778	Full	22	22	1300	600	1	Sandvik R390-044C4-11M060	4	44	90
		Down		13.5							
2	1789	Down		55	560	1233	1	Sandvik R390-125Q40-17H	11	125	90

**Table 2:** Natural frequencies and modal damping coefficients for identified (EMA) normal modes.

No.	Surface 1		Surface 2	
	Frequency [Hz]	Modal Damping [%]	Frequency [Hz]	Modal Damping [%]
1	172.1	0.996	91.1	9.342
2	187.3	2.083	113.1	9.936
3	233.0	1.618	150.8	4.542
4	253.6	0.859	173.2	0.777
5	265.0	1.458	196.5	0.972
6	280.4	0.902	279.2	1.572
7	373.1	1.908	307.5	1.225

**Table 3:** Milling features and adjusted parameters for the process simulation.

Milled Surface	Type of Milling	Milling Features				Adjusted Parameters				Simulation Time [min]
		*n* [rpm]	*v_c_* [m/min]	*v_f_* [mm/min]	RMS [mm]	*k_dl_* [daN/mm^2^]	*µ_l_*_2_	*µ_l_*_3_	Adjustment Time [min]	
1	Full:									
	standard	1300	180	600	0.000289	500	0.40	0.58	3	
	range	1300–1500	180–207	600–692		500	0.40	0.58		20
	the best	1500	207	692	0.000254	500	0.40	0.58		
2	Down:									
	standard	560	220	1233	0.002958	500	0.40	0.40	2	
	range	550–800	220–314	1211–1761		500	0.40	0.40		20
	the best	700	275	1541	0.000542	500	0.40	0.40		

**Table 4:** Spindle and feed speeds selected for milling the real workpiece.

Speed Selection	Surface 1			Surface 2		
	*a_p_* [mm]	Spindle Speed *n* [rev/min]	Feed Speed *v_f_* [mm/min]	*a_p_* [mm]	Spindle Speed *n* [rev/min]	Feed Speed *v_f_* [mm/min]
Standard	1	1300	600	1	560	1233
The best	1	1500	692	1	700	1541

**Table 5:** RMS values of displacements of performed milling operations for measurement points on the milled surfaces.

Surface 1							
Milling Type	Speed Selection	Displacements RMS [mm]					
		A22	A23	A32	A24	A25	Average
Full	Standard	0.000128	0.000340	0.000432	0.000390	0.000144	0.000287
	Standard - simulation -						0.000289
Down	Standard	0.000275	0.001376	0.001581	0.001281	0.000261	0.000955

Full	The best - prediction -						0.000254
	The best	0.000209	0.000318	0.000410	0.000364	0.000252	0.000311
Down	The best	0.000380	0.000545	0.000687	0.000592	0.000357	0.000512

**Surface 2**							
		A18	A19	A20	A21		Average
Down	Standard	0.003817	0.003105	0.002825	0.002113		0.002965
	Standard - simulation -						0.002958
Down	The best - prediction -						0.000542
	The best	0.001537	0.001474	0.001562	0.001539		0.001528

**Table 6:** Relative change in RMS values of displacements of performed milling operations for measurement points on the milled surfaces. The standard spindle speed milling is taken as the reference.

Surface 1							
Milling Type	The Best Spindle Speed Selection	Change in RMS Values [%]					
		A22	A23	A32	A24	A25	Average

Full	Prediction EAOVP						−12.1
	Milling EAOVP	63.3	−6.5	−5.1	−6.7	75.0	8.3
	Milling EAVP	35.9	−31.8	−25.9	−21.8	41.0	−13.9
Down	Milling EAOVP	38.2	−60.4	−56.5	−53.8	36.8	−46.4
	Milling EAVP	8.4	−63.7	−57.6	−54.4	25.3	−50.1

**Surface 2**							
		A18	A19	A20	A21		Average
Down	Prediction EAOVP						−81.7
	Milling EAOVP	−59.7	−52.5	−44.7	−27.2		−48.5
	Milling EAVP	−48.7	−37.2	7.6	−33.2		−42.2 −13.8

## Data Availability

The data presented in this study are available on reasonable request from the corresponding author.

## Appendix A

1. Inertia matrix of the structural subsystem:
  (A1) $$M=diag\left(m,\;m,\;m,J_{xr1},\;J_{xr2},\;J_{xr2}\right),$$
  m—mass of the RFE, $J_{xr1}$—mass moment of inertia with respect to the $x_{r1}$ axis of the RFE, $J_{xr2}$—mass moment of inertia with respect to the $x_{r2}$ axis of the RFE, and $J_{xr3}$—mass moment of inertia with respect to the $x_{r3}$ axis of the RFE.
2. Damping matrix of the structural subsystem:
  (A2) $$L=S_{rk}^{T}L_{k}S_{rk},$$
  **S***_rk_*_,_—matrix of coordinates of the SDE attachment point in local coordinate system *x_r_*_1_, *x_r_*_2_, and *x_r_*_3_ of the RFE,
  (A3) $$S_{rk}=\left[\begin{array}{cccccc} 1 & 0 & 0 & 0 & S_{rk3} & ‒S_{rk2}\\ 0 & 1 & 0 & ‒S_{rk3} & 0 & S_{rk1}\\ 0 & 0 & 1 & S_{rk2} & ‒S_{rk1} & 0\\ 0 & 0 & 0 & 1 & 0 & 0\\ 0 & 0 & 0 & 0 & 1 & 0\\ 0 & 0 & 0 & 0 & 0 & 1 \end{array}\right],$$
  $L_{k}=diag\left(l_{ki}\right),\;i=1,\;\ldots \;,\;6\;$—matrix of damping coefficients of the SDE.
3. Stiffness matrix of the structural subsystem:
  (A4) $$K=S_{rk}^{T}K_{k}S_{rk},$$
  $K_{k}=diag\left(k_{ki}\right),\;i=1,\;\ldots \;,\;6$—matrix of stiffness coefficients of the SDE.
4. Transformation matrix of displacements vector **q** from the *x_r_*_1_, *x_r_*_2_, and *x_r_*_3_ coordinates of the RFE, to the coordinate system *y_l_*_1_, *y_l_*_2_, and *y_l_*_3_ of CE no. *l*:
  (A5) $$T_{l}\left(t\right)=\Theta_{rl}\left(t\right)S_{rl}\left(t\right),$$
  (A6) $$\Theta_{rl}\left(t\right)=\left[\begin{array}{cc} \Theta_{rl}^{*}\left(t\right) & 0\\ 0 & \Theta_{rl}^{*}\left(t\right) \end{array}\right],$$
  $\Theta_{rl}^{*}\left(t\right)={\left[cos{\;}_{rlij}\left(t\right)\right]}_{3\times 3}$—matrix of direction cosines of time varying angles $\alpha_{rlij}\left(t\right)$ between axis $y_{li}$ of CE no. *l* and axis $x_{rj}$ of the RFE, i=1, …, 3, j=1, …, 3;
  $S_{rl}\left(t\right)$—matrix of variable-time coordinates of the attachment point of CE no. *l* in local coordinates system *x_r_*_1_, *x_r_*_2_, and *x_r_*_3_ of the RFE (similar to A3).
